# International High-Risk Clones Among Extended-Spectrum β-Lactamase–Producing *Escherichia coli* in Dhaka, Bangladesh

**DOI:** 10.3389/fmicb.2021.736464

**Published:** 2021-10-04

**Authors:** Razib Mazumder, Arif Hussain, Ahmed Abdullah, Md. Nazrul Islam, Md. Tuhin Sadique, S. M. Muniruzzaman, Anika Tabassum, Farhana Halim, Nasrin Akter, Dilruba Ahmed, Dinesh Mondal

**Affiliations:** ^1^Genomics Center, Laboratory Sciences and Services Division, International Centre for Diarrhoeal Disease Research, Bangladesh (icddr,b), Dhaka, Bangladesh; ^2^Clinical Microbiology and Immunology Laboratory, Laboratory Sciences and Services Division, International Centre for Diarrhoeal Disease Research, Bangladesh (icddr,b), Dhaka, Bangladesh

**Keywords:** CTX-M, NDM, MCR, extraintestinal pathogenic *E. coli* (ExPEC), carbapenem resistance, whole-genome sequencing, epidemiological successful clones, ST131 and non-ST131 lineages

## Abstract

**Background:** *Escherichia coli* is a major extended-spectrum β-lactamase (ESBL)–producing organism responsible for the rapid spread of antimicrobial resistance (AMR) that has compromised our ability to treat infections. Baseline data on population structure, virulence, and resistance mechanisms in *E. coli* lineages from developing countries such as Bangladesh are lacking.

**Methods:** Whole-genome sequencing was performed for 46 ESBL–*E. coli* isolates cultured from patient samples at the International Centre for Diarrhoeal Disease Research, Bangladesh (icddr,b)-Dhaka. Sequence data were analyzed to glean details of AMR, virulence, and phylogenetic and molecular markers of *E. coli* lineages.

**Results:** Genome comparison revealed presence of all major high-risk clones including sequence type 131 (ST131) (46%), ST405 (13%), ST648 (7%), ST410 (4.3%), ST38 (2%), ST73 (2%), and ST1193 (2%). The predominant ESBL gene and plasmid combination were *bla*_CTX__–__M__–__15_ and FII-FIA-FIB detected in diverse *E. coli* phylogroups and STs. The *bla*_NDM__–__5_ (9%) gene was present in prominent *E. coli* STs. One (2%) *mcr-1–*positive ST1011 *E. coli*, coharboring *bla*_CTXM__–__55_ gene, was detected. The extraintestinal pathogenic *E. coli* genotype was associated with specific *E. coli* lineages. The single nucleotide polymorphism (SNP)-based genome phylogeny largely showed correlation with phylogroups, serogroups, and *fimH* types. Majority of these isolates were susceptible to amikacin (93%), imipenem (93%), and nitrofurantoin (83%).

**Conclusion:** Our study reveals a high diversity of *E. coli* lineages among ESBL-producing *E. coli* from Dhaka. This study suggests ongoing circulation of ST131 and all major non-ST131 high-risk clones that are strongly associated with cephalosporin resistance and virulence genes. These findings warrant prospective monitoring of high-risk clones, which would otherwise worsen the AMR crises.

## Introduction

Infections with *Escherichia coli* that produce extended-spectrum β-lactamases (ESBLs) present an increasing clinical and public health threat (Mathers et al., 2015). These bacteria are resistant to several new-generation cephalosporin agents and render them ineffective for treating infections (Pitout and Laupland, 2008). Infections of ESBL *E. coli* causes increased morbidity, high mortality, longer hospital stays, and increased health care costs in comparison to infections caused by non-ESBL *E. coli* (Paterson and Bonomo, 2005). Treatment of such infections has been further complicated by the emergence of carbapenem resistance in ESBL–*E. coli*; these carbapenem-resistant isolates are often found resistant to all the available antibiotics (Doi, 2019).

Several strains of ESBL-producing *E. coli* are pathogenic; they commonly cause urinary tract infections (UTIs) in otherwise healthy people (Calbo et al., 2006; Franz et al., 2015). Moreover, when certain pathogenic bacterial clones horizontally acquire ESBL genes, they can emerge and reemerge rapidly within the population through clonal dissemination and thereby gain local or even global predominance as international high-risk clones (Price et al., 2013). Examples of such epidemiological successful extraintestinal pathogenic *E. coli* (ExPEC) lineages include sequence type 131 (ST131), ST410, ST38, ST73, ST405, and ST648, which are associated with both nosocomial and community-acquired infections and are being increasingly detected from multiple origins, worldwide (Baquero et al., 2013; Shaik et al., 2017; Manges et al., 2019). Since the last decade, CTX-M–type enzymes have become the most predominant ESBLs in *E. coli* and other Gram-negative bacteria of clinical significance (Cantón and Coque, 2006). Similarly, the transposable elements containing the New Delhi metallo-β-lactamase (NDM) gene have been identified to be responsible for the rapid dissemination of carbapenem resistance in *E. coli* (Kumarasamy et al., 2010).

Currently, a high number of ESBL-producing *E. coli* infections are linked to the pandemic *E. coli* lineage ST131 (Nicolas-Chanoine et al., 2008). Moreover, it is shown that *E. coli* ST131 strains are strongly associated with CTX-M-15–type ESBL and are predominantly responsible for causing bladder infections, kidney infections, and urosepsis worldwide including Southeast Asia (Nicolas-Chanoine et al., 2014; Chen et al., 2019). For instance, in India, it was reported that 93% of ST131 *E. coli* isolates carried CTX-M-15. Studies have also reported the presence of the *H30* subclone that is reportedly responsible for the clonal dissemination of ST131 *E. coli* (Hussain et al., 2012, 2014; Ranjan et al., 2015; Shaik et al., 2017). A study from Bangladesh reported that 71% of ESBL-producing *E. coli* was linked to the ST131 clone (Begum and Shamsuzzaman, 2016). Another study from Bangladesh identified a clonal cluster of clinical *E. coli* isolates belonging to serotype O25:H4, which indicates the widespread circulation of the ST131 *E. coli* lineage (Lina et al., 2014).

High-resolution studies on ESBL-producing *E. coli* strains from Bangladesh are lacking compared with the rest of the world, which limit our understanding of the population structure, emerging antimicrobial resistance (AMR) and their potential to spread. Herein, we report the findings of a genomic epidemiological investigation of the recent ESBL–*E. coli* isolates, especially the CTX-M–type ESBL producers associated with extraintestinal infections in Dhaka, Bangladesh. We analyzed their resistance genes, virulence genes, plasmid types, phylogenetic relatedness, and molecular features. Our study suggests ongoing circulation of ST131 and all major non-ST131 *E. coli* high-risk clones in Dhaka, Bangladesh. Studies such as these will provide important insights into the evolution of pathogens and will inform novel interventional strategies.

## Materials and Methods

### Study Setting, Bacterial Isolates, and Antimicrobial Susceptibility

Bacterial isolates were collected randomly as part of routine (1%) surveillance (128 *E. coli* isolates) between March 2018 and July 2019 from the Clinical Microbiology and Immunology Laboratory of the International Center for Diarrheal Disease Research, Bangladesh (icddr,b) (Mazumder et al., 2020a). From this collection of 128 *E. coli* isolates, 46 randomly selected ESBL *E. coli* spanning over the entire 16-month period (representing 1–4 isolates per month) were whole genome sequenced. Of these 46 isolates, 34 were obtained from urine and 12 from pus specimen (Table 1). Identification of bacterial isolates was performed using standard biochemical methods. Antimicrobial susceptibility testing by disk diffusion was performed against the following antibiotics: gentamicin (10 μg), amikacin (30 μg), cotrimoxazole (25 μg), ciprofloxacin (5 μg), nitrofurantoin (200 μg), cefuroxime (30 μg), ceftriaxone (30 μg), cefixime (5 μg), cefepime (30 μg), and imipenem (10 μg). *E. coli* ATCC 25922 was used for quality control (Clinical and Laboratory Standards Institute, 2015).

**TABLE 1:** Genome features and metadata of 46 ESBL *E. coli* isolates sequenced in this study.

**Sl no.**	**Isolate ID**	**Date of isolation**	**Source**	**STs**	**Genome coverage**	**Accession no.**	**Contig no. (>200 bp)**	**Genome size (bp)**	**No. of Coding Sequences (CDS)**
1	LMLEEc001	20-Apr-2018	Urine	131	83.9X	JACHQR000000000.1	104	*5,220,525*	4,897
2	LMLEEc002	3-May-2018	Urine	3,748	91.4X	JACHQQ000000000.1	160	5,079,033	4,793
3	LMLEEc003	3-May-2018	Pus	131	91.4X	JACHQP000000000.1	199	5,210,796	4,887
4	LMLEEc010	2-Jun-2018	Urine	131	95.4X	JACHQO000000000.1	112	5,299,824	5,029
5	LMLEEc018	28-Jun-2018	Pus	410	92.3X	JACHQN000000000.1	98	4,888,814	4,570
6	LMLEEc019	28-Jun-2018	Pus	410	97.8X	JACHQM000000000.1	99	4,888,382	4,566
7	LMLEEc020	5-Jul-2018	Urine	167	88.6X	JACHQL000000000.1	150	4,963,734	4,655
8	LMLEEc025	19-Jul-2018	Urine	131	113.5X	JACHQK000000000.1	119	5,076,042	4,785
9	LMLEEc027	26-Jul-2018	Urine	131	62.9X	JACHQJ000000000.1	123	5,211,152	4,905
10	LMLEEc029	2-Aug-2018	Urine	131	67X	JACHQI000000000.1	97	5,234,461	4,966
11	LMLEEc033	16-Aug-2018	Urine	162	71.4X	JACHQH000000000.1	113	5,004,684	4,677
12	LMLEEc034	24-Aug-2018	Urine	131	71.4X	JACHQG000000000.1	113	5,261,112	4,939
13	LMLEEc035	23-Aug-2018	Pus	648	65.4X	JACHQF000000000.1	188	5,376,792	5,053
14	LMLEEc036	31-Aug-2018	Urine	131	73.2X	JACHQE000000000.1	86	5,237,487	4,928
15	LMLEEc039	6-Sep-2018	Urine	648	83.2X	JACHQD000000000.1	118	5,119,774	4,741
16	LMLEEc040	13-Sep-2018	Urine	131	65.6X	JACHQC000000000.1	122	5,263,046	4,943
17	LMLEEc041	13-Sep-2018	Urine	1,193	73.7X	JACHQB000000000.1	82	5,023,339	4,696
18	LMLEEc043	20-Sep-2018	Urine	131	62.1X	JACHQA000000000.1	92	4,972,440	4,633
19	LMLEEc050	18-Oct-2018	Urine	131	54.6X	JACHPZ000000000.1	92	5,237,567	4,926
20	LMLEEc056	8-Nov-2018	Urine	405	62.2X	JACHPY000000000.1	149	5,278,053	4,880
21	LMLEEc059	15-Nov-2018	Urine	405	66.8X	JACHPX000000000.1	156	5,348,292	4,939
22	LMLEEc062	29-Nov-2018	Urine	405	67.4X	JACHPW000000000.1	129	5,228,791	4,886
23	LMLEEc063	29-Nov-2018	Urine	131	62.5X	JACHPV000000000.1	83	5,180,621	4,908
24	LMLEEc064	6-Dec-2018	Urine	2,851	53X	JACHPU000000000.1	156	5,180,445	4,969
25	LMLEEc070	29-Dec-2018	Pus	2,178	83.2X	JACHPT000000000.1	90	4,980,462	4,708
26	LMLEEc071	29-Dec-2018	Pus	131	73.1X	JACHPS000000000.1	65	5,110,507	4,817
27	LMLEEc072	3-Jan-2019	Urine	131	76.9X	JACHPR000000000.1	113	5,248,087	4,933
28	LMLEEc074	10-Jan-2019	Urine	131	89.8X	JACHPQ000000000.1	121	5,315,415	5,069
29	LMLEEc078	24-Jan-2019	Urine	421	143.1X	JACHPP000000000.1	106	5,087,660	4,788
30	LMLEEc082	14-Feb-2019	Urine	131	89.8X	JACHPO000000000.1	109	5,282,720	5,015
31	LMLEEc087	21-Feb-2019	Urine	131	67.9X	JACHPN000000000.1	115	5,197,822	4,882
32	LMLEEc088	28-Feb-2019	Urine	648	73.9X	JACHPM000000000.1	135	5,193,220	4,906
33	LMLEEc089	28-Feb-2019	Urine	405	62.7X	JACHPL000000000.1	166	5,359,628	5,003
34	LMLEEc091	6-Mar-2019	Urine	617	50.6X	JACHPK000000000.1	143	5,042,428	4,717
35	LMLEEc097	28-Mar-2019	Urine	131	68.2X	JACHPJ000000000.1	125	5,354,581	5,109
36	LMLEEc101	11-Apr-2019	Pus	48	77.9X	JACHPI000000000.1	121	4,535,056	4,220
37	LMLEEc103	18-Apr-2019	Pus	131	85X	JACHPH000000000.1	106	5,233,284	4,952
38	LMLEEc104	25-Apr-2019	Urine	131	69.4X	JACHPG000000000.1	100	5,277,281	5,004
39	LMLEEc108	9-May-2019	Urine	405	62.4X	JACHPF000000000.1	173	5,515,065	5,131
40	LMLEEc111	23-May-2019	Urine	38	63.4X	JACHPE000000000.1	93	4,982,213	4,587
41	LMLEEc115	13-Jun-2019	Pus	73	78.7X	JACHPD000000000.1	138	5,149,754	4,752
42	LMLEEc117	7-Jun-2019	Urine	354	56.4X	JACHPC000000000.1	93	5,346,716	5,078
43	LMLEEc120	27-Jun-2019	Urine	131	49.8X	JACHPB000000000.1	159	5,245,925	4,948
44	LMLEEc123	6-Jul-2019	Pus	1,011	79X	JACHPA000000000.1	140	5,218,770	4,823
45	LMLEEc125	11-Jul-2019	Pus	405	76.5X	JACHOZ000000000.1	216	5,514,459	5,180
46	LMLEEc127	20-Jul-2019	Pus	1,884	68.2X	JACHOY000000000.1	72	5,050,573	4,666

### Whole-Genome Sequencing, Genome Assembly, and Annotation

Genomic DNA from an overnight bacterial culture was extracted and purified using QIAamp DNA Mini kit (Qiagen, Germany). The purity of the genomic DNA was assessed using a NanoDrop spectrophotometer (Thermo Fisher Scientific, United States), and quantification was performed using a Qubit 2.0 fluorometer (Life Technologies). DNA libraries were prepared using the Nextera XT DNA library Prep kit (Illumina) (Mazumder et al., 2020b). The pooled library was sequenced at the genomic sequencing facility of icddr,b on an Illumina Nextseq500 system using the 150-base-paired-end Mid-output v2.5 sequencing kit. The sequence reads were trimmed and filtered using Trimmomatic 0.39 (Bolger et al., 2014), and *de novo* assemblies of the resulting reads were generated using SPAdes 3.11.1 (Bankevich et al., 2012), and the quality check was done using QUAST v5.02 (Mikheenko et al., 2018). The genomes of our study were annotated using Prokka *de novo* (Seemann, 2014). The program was run on fast mode, and the genus-specific BLAST database was used. The genome features and associated metadata are listed in Table 1.

### *In silico* Molecular Analysis

Phylotyping was done using the ClermonTyping online tool (Beghain et al., 2018). Other molecular features were identified using the Center for Genomic Epidemiology’s Bioinformatics tools using default parameters unless otherwise stated. Specifically, MLST 2.0 (Larsen et al., 2012) was used to identify STs, SerotypeFinder 1.1 (Joensen et al., 2015) for detecting serotypes, FimTyper 1.0 (Roer et al., 2017) to determine *fimH* types, and PlasmidFinder 2.1 (Carattoli et al., 2014) for identification of plasmid incompatibility groups. Comprehensive Antibiotic Resistance Database (McArthur et al., 2013) was used for screening the presence of acquired AMR genes; virulence genes were detected using a custom-made database of 50 genes from the Virulence Factors Database (Chen et al., 2005) belonging to different categories as described previously (Stoesser et al., 2015), a threshold, ≥ 90% identity; minimum coverage, ≥ 70% was used for both. AMR-related chromosomal point mutations were identified in the draft genomes using PointFinder (Zankari et al., 2017). The genetic context of *bla*_CTX–M–15_, *bla*_NDM–5_, and *mcr-1* with respect to plasmid was determined by BLAST searching of particular contigs against the GenBank database. Genomes having three or more ExPEC-associated genes were classified as ExPEC according to Johnson’s criteria (Johnson and Stell, 2000). From virulence and resistance gene presence and absence CSV files, heat plots were obtained, using the Python module seaborn and matplotlib.

### Single Nucleotide Polymorphism-Based Core Genome Phylogeny

The reference (*E. coli* 0154:H7 Sakai strain) guided multi-fasta consensus alignment of 46 *E. coli* genomes was obtained using the Snippy v4.4.0 pipeline (Seemann, 2015). Putative recombination regions were detected and masked using Gubbins v2.3.4 (Croucher et al., 2015). Finally, the SNP- based phylogeny was inferred using RaxML v8.2.12 (Stamatakis, 2014) using GTR (Generalized Time Reversible) substitution model and GAMMA distribution as the model of rate heterogeneity.

### Statistical Analysis

Using SPSS statistics software (version 25.0), continuous variables were compared with the non-parametric Mann–Whitney *U-*test, and proportions were analyzed using the χ^2^-test; *p*-values with a threshold of < 0.05 were considered statistically significant. Accordingly, the *p*-values are indicated within text where appropriate.

## Results

### Molecular Typing of Extended-Spectrum β-Lactamase *Escherichia coli* Isolates

**FIGURE 1:**
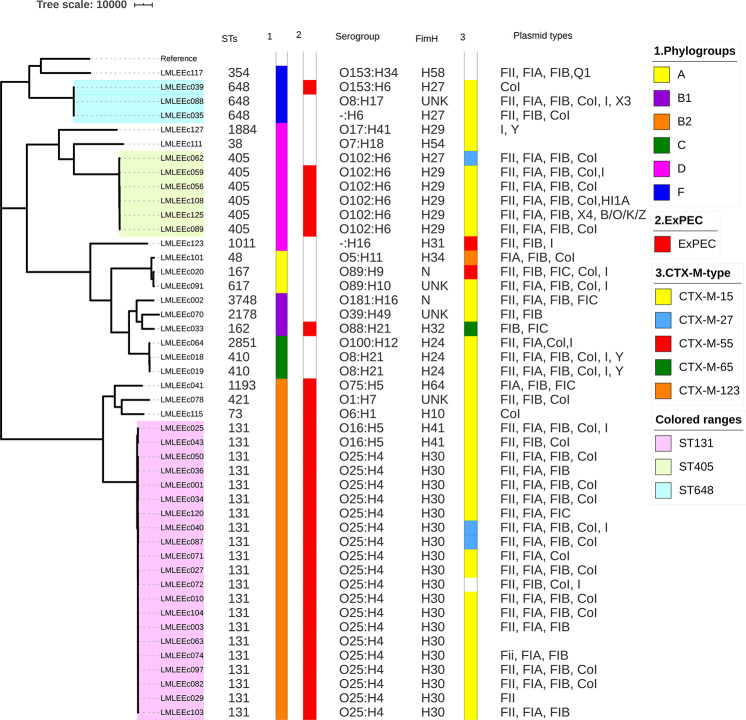
Core-genome SNP-based phylogenetic tree representing the population structure and comparison of genetic traits observed in 46 ESBL *E. coli* isolates. Sequence types, phylogroups, ExPEC status, serogroups, *fimH* types, CT-X-M types, and plasmid replicon types are shown next to the tree.

The sequence diversity of the study isolates as analyzed by *in silico* MLST demonstrates that the 46 ESBL–*E. coli* isolates belonged to 19 different STs (Figure 1). The pandemic ST131 lineage was the most predominant ST identified, comprising 46% (21/46) of the isolates. Apart from ST131, the other significant STs included the presence of isolates belonging to the international high-risk clones, such as ST405 (13% 6/46), ST648 (7% 3/46), ST410 (4.3% 2/46) ST38 (2%), ST73 (2%), and ST1193 (2%). Phylogrouping identified a total of six phylogroups, namely, A, B1, B2, C, D, and F, in the ESBL–*E. coli* isolates (Figure 1). The phylogroup B2 (24/46) was the major phylogroup in our collection, which predominantly consisted of ST131 isolates (88%, 21/24). Phylogroup D was the second largest phylogroup with 20% isolates (*n* = 9, 6 ST405, 1 ST38, 1 ST1011, 1 ST1884), which was followed by group F (*n* = 4, 3 ST648, 1 ST354), B1 (*n* = 3, 1 ST3748, 1 ST162, 1 ST2178), C (*n* = 3, 2 ST410, 1 ST2851), and A (*n* = 3, 1 ST167, 1 ST617, 1 ST48). Serotyping revealed that out of the 21 ST131 *E. coli*, 19 had serotype O25:H4, and two had O16:H5 serogroup (Figure 1). All 19 ST131 *E. coli* with O25; H4 serogroup had *fimH* 30 alleles, whereas the two ST131 *E. coli* isolates with O16:H5 serogroup had *fimH*41 allele, whereas the non-ST131 isolates exhibited diverse serotypes that included 19 different serotypes. Among them, the six emerging urosepsis pathogenic isolates belonging to ST405 had O102:H6 serogroup with *fimH* 29 allele except for one strain, which had *fimH* 27. The two isolates of high-risk clone ST410 had O8:H21 serogroup with *fimH* 24 allele. However, the three isolates belonging to the emerging pandemic lineage ST648 were not associated with any distinct sero or *fimH* group. Similar to the results of serogroups, the non-ST131 isolates exhibited diverse *fimH* (10 different) types.

### Phenotypic and Genotypic Antimicrobial Resistance

**FIGURE 2:**
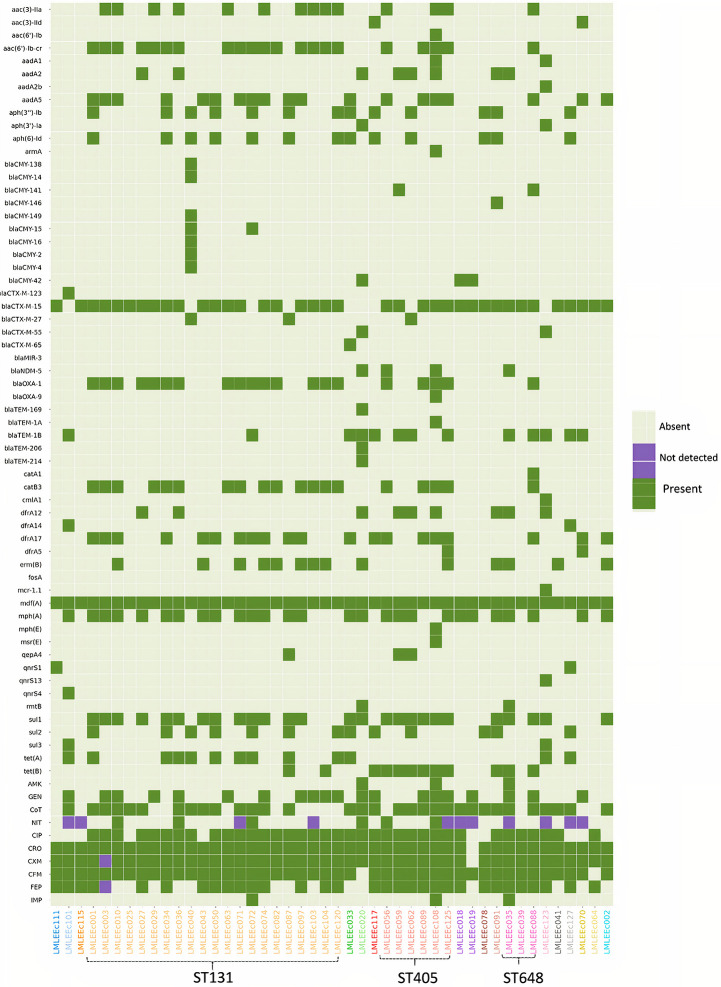
Heatmap showing the distribution of acquired AMR genes and AMR phenotypic profiles within 46 *E. coli* isolates. Gene names are listed on the left, and strain names are listed below the image. The presence of gene is indicated by colored blocks, and the gene absence is indicated by gray blocks. CRO, ceftriaxone; CXM, cefuroxime; CFM, cefixime; FEP, cefepime; NIT, nitrofurantoin; AMK, amikacin; CIP, ciprofloxacin; CoT, cotrimoxazole; IMP, imipenem; GEN, gentamicin.

Majority of isolates were susceptible to amikacin (93%), imipenem (93%), and nitrofurantoin (83%) (Figure 2). On the other hand, a majority of isolates were resistant to the common, empirically used antibiotics such as cefixime (96%), cefuroxime (96%), ceftriaxone (98%), and ciprofloxacin (78%). Besides, a moderate number of isolates were also resistant to cotrimoxazole (70%), gentamicin (43%), and cefepime (67%). In contrast to the non-ST131 *E. coli*, isolates belonging to the ST131 lineage had higher resistance prevalence as the aggregate resistance score [median (range)] for ST131 isolates [6 (4–8)] was higher than that of non-ST131 isolates [5.5 (0–10)]. However, the difference was not statistically significant (*p* > 0.5 Mann–Whitney *U*-test).

Whole-genome sequencing (WGS) analysis identified 60 acquired AMR genes that are known to encode proteins conferring resistance to different classes of antibiotics including β-lactams, aminoglycosides, chloramphenicol, tetracycline, sulfonamides, trimethoprim, fluoroquinolone, and colistin (Figure 2).

#### β-Lactam and Extended-Spectrum β-Lactamase Resistance

We identified 24 genes associated with β-lactam resistance including 13 ESBL, 10 AmpC β-lactamase, and 1 carbapenemase gene. The 13 ESBL genes included *bla*_OXA__–__1_ (43%), *bla*_TEM__–1B_ (28%), *bla*_CTX__–__M__–__27_ (7%), and *bla*_CTX__–__M__–__55_ (4%). Thirty-eight of 46 isolates (81%) carried the *bla*_CTX__–__M__–__15_ gene. Of 38 isolates carrying the *bla*_CTX__–__M__–__15_ gene, 36 were resistant to ceftriaxone and cefixime; therefore, this gene was strongly associated with third-generation cephalosporin resistance (*p* < 0.001). Moreover, the gene *bla*_CTX__–__M__–__15_ was detected in isolates of diverse genetic backgrounds affiliated to 6 different phylogroups and 13 different STs, including the high-risk clones—ST131 (18/21), ST405 (5/6), ST648 (3/3), ST410 (2/2), ST38 (1/1), ST73 (1/1), and ST1193 (1/1). The *bla*_CTX__–__M__–__15_ gene was located on a plasmid for 23 of 38 isolates. Isolates also harbored other ESBL genes that included *bla*_CTX__–__M__–__123_, *bla*_CTX__–__M__–__65_, *bla*_MIR__–__3_, *bla*_OXA__–__9_, *bla*_TEM__–__169_, *bla*_TEM__–1A_, *bla*_TEM__–1B_, *bla*_TEM__–__206_, and *bla*_TEM__–__214_ in lesser proportion. Similarly, a low prevalence of AmpC β-lactamase genes was detected (Figure 2).

#### Aminoglycoside Resistance

We identified 12 genes known to confer resistance to aminoglycosides (Figure 2). These included *aac(6′)-Ib-cr* (45%), *aadA5* (40%), *aac(3)-IIa* (30%), *aph(3′′)-Ib* (30%), *aph(6)-Id* (30%), and *aadA2* (17%). Of these six prominent aminoglycoside genes, only two, *aac(3)-IIa* (14/13) and *aac(6′)-Ib-cr* (21/14), were strongly associated with gentamicin resistance (*p* < 0.001). None of the aminoglycoside genes was associated with amikacin resistance. Approximately 4% of the isolates harbored *aac(3)-IId*, *aadA1*, and *aph(3′)-Ia*; other genes of this class included *aac(6′)-Ib* (one isolate), *aadA2b* (one isolate), and *armA* (one isolate).

#### Cotrimoxazole Resistance

Three genes known to encode sulfonamide resistance were identified: *sul1* in 26/46 (57%) isolates, *sul2* in 14/46 (30%) isolates, and *sul3* in 2/46 (4%) isolates. The presence of *sul1* was positively correlated with phenotypic resistance to cotrimoxazole (23/26) (*p* < 0.001). Similarly, of the four genes responsible for trimethoprim resistance [*dfrA17* (43%), *dfrA12* (19%), *dfrA14* (4%), *dfrA5* (4%)], the gene dfrA17 was strongly associated with cotrimoxazole resistance (*p* < 0.001).

#### Fluoroquinolone Resistance

Using PointFinder, we identified two mutations in *gyrA* including S83L (serine to leucine) and D87N (aspartic acid to asparagine). The mutation S83L was detected in 13% (6/46) of isolates. In contrast, 80% (37/46) of isolates had both the mutations (S83L and D87N); the dual *gyrA* mutants were all resistant to ciprofloxacin. However, isolates (4/46) having a single mutation (S83L) were all susceptible to ciprofloxacin. Similarly, in the *parC* amino acid product, we identified two substitutions at codon position 80 (serine to isoleucine) and codon 84 (glutamic acid to valine). The mutation S80I was present in 33% (15/46) of isolates, and the mutation E84V was detected in one isolate, whereas 22 of 46 isolates harbored both of these mutations, and they were all resistant to ciprofloxacin. Three substitutions were also detected in *parE* gene, with I529L being predominant (20/46) followed by S458A (11/46) and L416F (1/46). All isolates (20/46) carrying I529L mutation were ciprofloxacin-resistant, and this mutation was found in the majority of ST131 strains (18/21 ST131 strains). Additionally, the plasmid-mediated quinolone resistance gene *qnrs*1 was detected in two isolates followed by *qnrS*4 and *qnrS*13 in one isolate each. Except for *qnrs*13, isolates carrying *qnrS* genes were susceptible to ciprofloxacin.

#### Carbapenem and Colistin Resistance

NDM-5 carbapenemase was detected in four *E. coli* isolates (8.5%). All four isolates belonged to the prominent clonal groups: two ST405 (phylogroup D), one ST648 (phylogroup F), and one ST167 (phylogroup A). All four *bla*_*NDM–5*_ genes were found to be located on plasmids (4/4 isolates). Except for one (ST167), all three isolates coharbored a *bla*_CTX__–__M__–__15_ gene. Phenotypic resistance to imipenem was detected in only two of four *bla*_NDM__–__5_–positive isolates. Nonetheless, all four *bla*_NDM__–__5_–positive *E. coli* were pan–drug-resistant as resistance was detected to at least 7 of 10 antibiotics tested. We detected one *mcr-1*–positive *E. coli* belonging to ST1011; the *mcr-1* gene was detected on a plasmid. This strain was coharboring a *bla*_CTXM__–__55_ gene and showed phenotypic resistance to cotrimoxazole, ciprofloxacin, ceftriaxone, cefuroxime, and cefixime and was notably susceptible to imipenem, gentamicin, and amikacin.

### Replicon Typing

Screening of plasmid replicons among 46 *E. coli* isolates using PlasmidFinder database detected a total of 12 plasmid replicons, which included FII, FIA, FIB, FIC, Col, I, X3, X4, Q1, B/O/K/Z, HI, and Y (Figure 1). FII was the predominant replicon identified in 83% (38/46) of isolates followed by FIB, Col, and FIA replicon types, with 80% (37/46), 72% (33/46), and 70% (32/46), respectively. The *mcr-1–*positive *E. coli* harbored FII and FIB plasmid replicon types.

### Virulence Genes

**FIGURE 3:**
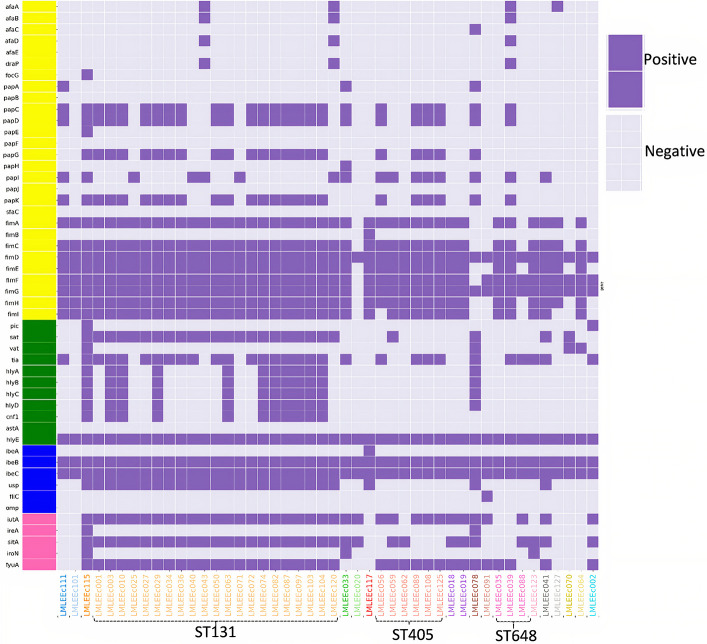
Distribution of virulence gene profiles within 46 *E. coli* isolates are represented in the heatmap, with colored blocks indicating the gene presence and gray-colored blocks indicating the gene absence. Further, the virulence genes are vertically colored coded by functional categories (yellow, adhesins; blue, toxins; pink, miscellaneous; green, siderophores).

Figure 3 shows the distribution of virulence genes among the 46 sequenced *E. coli* isolates. A total of 32 of the 46 *E. coli* isolates were classified as ExPEC, which comprised 21/21 ST131 isolates (100%) and 11/26 non-ST131 isolates (42%). The ExPEC isolates identified were enriched with virulence genes such as *pap*, *fim*, *sat*, *tia*, *hlyE*, *iutA*, *sitA*, and *fyuA.* Isolates belonging to the ST131 clone showed higher aggregate virulence scores [median (range)], 22 (18–27) compared with non-ST131 isolates 14.5 (6–33). The only isolate that carried the highest virulence genes (33) belonged to ST73.

### Population Structure of Extended-Spectrum β-Lactamase *Escherichia coli* Isolates

To investigate the relatedness of 46 ESBL *E. coli* isolates, single nucleotide polymorphism (SNP)-based core genome phylogenetic tree was constructed (Figure 1). The 46 *E. coli* genomes clustered with a good concordance to their phylogroups. Particularly, strains belonging to phylogroups B2, D, and F formed distinct clades, whereas strains belonging to phylogroups A, B1, and C were clustered together under a large clade; 24 of these 46 *E. coli* isolates formed a large cluster of B2 phylogroup all qualified as ExPEC and had the most common plasmid combination of FII, FIA, and FIB; this clade mainly comprised (21/24) ST131 isolates. Further, it was identified that 16 of 21 ST131 *E. coli* belonged to the most virulent, widespread H30Rx subclone, three belonged to H30R, and two belonged to fluoroquinolone-susceptible *fimH 41*, CTX-M-15–positive ST131 *E. coli*. The 21 ST131 *E. coli* isolates showed little or no difference in the SNP analysis; these isolates were collected over 15 months, which indicates widespread dissemination of this particular lineage. The sister clade of ST131 clustered *E. coli* lineages that included ST1193, ST73, and ST421. When the branches of non-ST131 isolates were assessed closely, high clonality was observed between individual isolates in a few cases. For instance, six strains of ST405 formed an identical cluster; these were collected from November 2018 to July 2018, and five of six isolates carried *bla*_CTX__–__M__–__15_ and had identical serogroup and *fimH* types with almost common plasmid profile. However, two of these six isolates carried *bla*_NDM__–__5_ gene in addition to the shared profiles. Similarly, ST410 isolates formed identical clusters with shared molecular profiles. However, ST648 strains formed an identical cluster but demonstrated diverse molecular profiles.

## Discussion

ESBL-producing *E. coli* is responsible for a significant number of nosocomial and community-acquired infections (Pana and Zaoutis, 2018). In this study, we used a WGS approach to analyze 46 ESBL–*E. coli* isolates collected over a year (16 months) from a referral diagnostic center in Dhaka, Bangladesh, a Southeast Asian country from which data such as these are acutely lacking.

Our study revealed that the ST131 lineage is by far the most prevalent lineage (45%) represented particularly by H30Rx isolates (34%). This dominance of ST131 *E. coli* in our study isolates is possibly because of our focus on ESBL–*E. coli*. However, the non-ST131 (55%) *E. coli* isolates showed high diversity with respect to phylogroups, phylogenetic clustering, STs, serotypes, and *fimH* types, which is consistent with previous reports (Hussain et al., 2014; Musicha et al., 2017). Furthermore, we demonstrated that ESBLs, in particular, the CTX-M–type ESBLs, in Bangladesh have emerged across major high-risk international clones that comprise ST38, ST73, ST405, ST410, ST648, and ST1193 lineages, in addition to isolates of the pandemic *E. coli* ST131.

Molecular typing revealed that a majority of CTX-M–type ESBL producers belonged to the ST131 lineage, which constituted the most clonal group in the population structure of the studied isolates. The predominance of this lineage and its association with CTX-M-15 is in line with the reports from worldwide studies (Nicolas-Chanoine et al., 2014), including those from Asia (Hussain et al., 2012; Ranjan et al., 2015). Further, we documented a greater proportion (76%) of H30Rx subclones within the ST131 *E. coli* isolates, indicating a scenario of endemic circulation of H30Rx strains in the current setting, as these strains have been recovered between the span of April 2018 to June 2019. Clonal expansion of H30RX subclone might be the reason for the high prevalence and transmission of CTX-M-15–associated ST131 *E. coli* isolates in this setting. The H30Rx subclone is also reported in the neighboring country, India, in 2015 (Ranjan et al., 2015). The H30Rx isolates detected in this study were all qualified as ExPEC, which harbored a broad range of virulence genes with a relatively high virulence score, median (range): 27 (18–27). These isolates exhibited a high prevalence of a combination of IncFII, IncFIA, and IncFIB plasmid replicons, which is consistent with a recent WGS study from North Carolina (Kanamori et al., 2017). The gene *aac(6’)-lb-cr* encoding the aminoglycoside and fluoroquinolone-modifying enzyme was strongly associated with H30Rx strains. In addition to the *bla*_CTX__–__M__–__15_ gene, they exhibited a high prevalence of *bla*_OXA__–__1_ and high resistance to third-generation cephalosporin antibiotics (ceftriaxone and cefixime). The association of H30Rx subclone with extended-spectrum cephalosporin and fluoroquinolone resistance might have contributed to their predominance and epidemiological success over other *E. coli* lineages.

In this study, we have further shown that the *bla*_CTX__–__M__–__15_ gene exists in genetically diverse strains including the prominent non-ST131 uropathogenic *E. coli* lineages such as ST38, ST73, ST405, ST410, ST648, and ST1193. Isolates of ST38 were reported to be evolving and is described to be increasingly detected in UTIs, which was previously considered to be a gut pathogen because they harbor genes for both EAEC and ExPEC (Chattaway et al., 2014). It is noticed that the ST38 isolate identified in this study was moderately lower in virulence and AMR gene content; particularly, it lacked the fluoroquinolone resistance and plasmids. Nonetheless, they were identified as ExPEC. ST73 is another frequently isolated ExPEC from UTI and bloodstream infections (Riley, 2014). The ST73 isolate in our study was found to carry an extensive array of virulence genes, whereas it was susceptible to most of the antibiotics and carried only IncCoI plasmid type, in contrast to other studies, which detected FII, FIA, and FIB plasmids; the reason for this could be that the ST73 strains are not expanding clonally as also suggested by others (Bogema et al., 2020).

ST405 *E. coli* lineage has been implicated as drivers of *bla*_CTXM__–__15_ and is often associated with *bla*_NDM_ genes and extensive virulence repertoire similar to the ST131 (Devanga Ragupathi et al., 2020). All six ST405 isolates from our study demonstrated high AMR rates and specifically carried a set of common plasmid replicons, including IncFII, IncFIA, IncFIB, and IncCoI. Five of these six ST405 strains qualified as ExPEC with extensive virulence gene profiles, consistent with other reports (Zhang et al., 2018; Devanga Ragupathi et al., 2020). Two of these six ST405 isolates also harbored *bla*_NDM__–__5_ gene. ST410 is considered another emerging “high-risk” clone that is resistant to fluoroquinolones, cephalosporins, and sometimes carbapenems (Roer et al., 2018). The two ST410 isolates in our collection showed moderate virulence and resistance profiles; they harbored *bla*_CTX__–__M__–__15_ gene with a set of five plasmid replicon types and *fimH* 24 allele (Figure 1). The lineage ST648 has been predicted to become another internationally circulating clone that will worsen infection treatment possibilities because of its AMR (Schaufler et al., 2019). The three ST648 isolates detected in this study carried *bla*_CTX__–__M__–__15_ and demonstrated high AMR rates; all the three strains consistently carried IncCoI plasmids. One ST648 strain was also positive for the *bla*_NDM__–__5_ gene. ST1193 is the latest pandemic multidrug-resistant uropathogenic clonal group (Johnson et al., 2019), which is usually detected among non-lactose fermenters (Johnson et al., 2019). The ST1193 isolate in our collection was qualified as ExPEC; it showed high resistance rates, with particular resistance to fluoroquinolones, cephalosporins, and cotrimoxazole. It harbored IncFIA, IncFIB, and IncCoI plasmid types.

Several WGS studies have described the features of *E. coli* ST131. In contrast, not many have addressed the molecular epidemiology of non-ST131 *E. coli* worldwide. In this study, we have described that the ST131 *E. coli* is currently the most prevalent CTX-M–type ESBL producer in our community. However, we have also drawn attention to the emergence of significant clonal groups among non-ST131 *E. coli*. Further work is warranted in Bangladesh to accurately estimate the prevalence of these clonal groups and systematically analyze them in a global context, which is pertinent from public health and clinical standpoint.

Taken together, our study suggests that the CTX-M–type ESBLs and particularly the CTX-M-15 are prevalent among diverse *E. coli* STs circulating in Dhaka, Bangladesh. Further, our findings confirm the striking predominance of ST131 lineage and also revealed the presence of several major high-risk non-ST131 ExPEC clonal lineages, such as ST38, ST73, ST405, ST410, ST648, and ST1193, all associated with cephalosporin resistance and virulence. Further genomic epidemiological studies are needed to keep track of significant virulent/multiresistant *E. coli* clones in Bangladesh. Such studies are needed to inform us about the evolutionary dynamics of pathogenicity and resistance in emerging *E. coli* lineages of public health concern which will aid in evidence-based infection control and antibiotic-prescribing policies.

## Data Availability Statement

The datasets generated for this study can be found in the GenBank (Bioproject Accession: PRJNA654992). The GenBank accession numbers of 46 genomes sequenced in this study can be found in Table 1.

## Author Contributions

AH and RM designed the study, performed genome sequencing, and drafted the manuscript. AA, AH, and RM carried out the bioinformatics analyses and interpretation of results. MI, MS, SM, AT, FH, and NA were involved in the culture and AST of bacteria isolates. DA and DM supervised the study. All authors have read and approved the final manuscript.

## Conflict of Interest

The authors declare that the research was conducted in the absence of any commercial or financial relationships that could be construed as a potential conflict of interest.

## Publisher’s Note

All claims expressed in this article are solely those of the authors and do not necessarily represent those of their affiliated organizations, or those of the publisher, the editors and the reviewers. Any product that may be evaluated in this article, or claim that may be made by its manufacturer, is not guaranteed or endorsed by the publisher.
